# Prolonged severe neutropenia after the first daratumumab administration for multiple myeloma with baseline neutropenia

**DOI:** 10.1007/s00277-019-03711-0

**Published:** 2019-05-16

**Authors:** Fumihiko Nakamura, Ryo Nasu

**Affiliations:** Department of Hematology, National Center for Global Health and Medicine, 1-21-1 Toyama, Shinjuku-ku, Tokyo, 162-8655 Japan

Dear Editor,

Daratumumab, an anti-CD38 monoclonal antibody, shows substantial efficacy for relapsed and refractory multiple myeloma (MM). Daratumumab also has a clinically manageable and acceptable safety profile. Grade 3–4 neutropenia was recorded in 5–12% of patients receiving single-daratumumab according to phase 1–2 trials [1, 2]. Although neutropenia was more common in daratumumab triplet therapy, the addition of daratumumab did not significantly increase documented infections and treatment discontinuation [3, 4]. Importantly, patients with absolute neutrophil count (ANC) of 1 × 10^9^/L or less were excluded from these clinical trials. The efficacy and safety profiles in patients with baseline low ANC have not yet been elucidated. Herein, we report a unique case showing prolonged severe neutropenia after the first daratumumab dose.

**Fig. 1:**
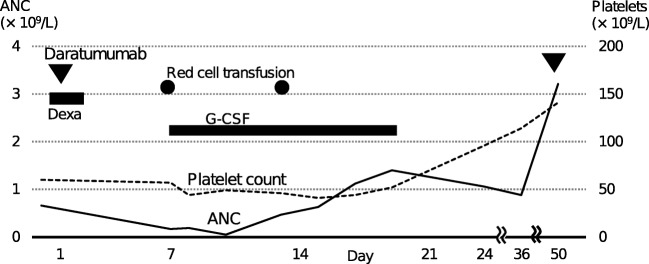
Clinical course after the first daratumumab administration. Closed triangle and circle indicate daratumumab infusion and red cell transfusion, respectively. ANC, absolute neutrophil count; Dexa, dexamethasone; G-CSF, granulocyte-colony stimulating factor

A 70-year-old Japanese woman was found to have pancytopenia at a medical checkup and was diagnosed as MM. Very good partial response with complete resolution of cytopenias was achieved by bortezomib plus dexamethasone therapy. At the age of 73 years, increasing paraprotein concentration and progressive pancytopenia were noted during second-line therapy with ixazomib, lenalidomide, and dexamethasone. Bone marrow trephine biopsy then disclosed a hypoplastic, fatty marrow with an increase in myeloma cells and suppression of normal hematopoiesis. Treatment was changed to daratumumab plus dexamethasone therapy. Neither lenalidomide nor bortezomib was added because pretreatment ANC and platelet count were 0.66 and 60 × 10^9^/L, respectively. Daratumumab at a dose of 16 mg/kg was administered without any infusion-related reactions. Six days later, ANC dropped to 0.17 × 10^9^/L while platelet count remained unchanged (Fig. 1). Granulocyte-colony stimulating factor (G-CSF) and prophylactic levofloxacin were initiated. After reaching a nadir, not only ANC but also hemoglobin level and platelet count gradually improved. On day 50, blood counts returned to normal and daratumumab administration was resumed in a 28-day cycle. Hematologic adverse events never occurred during the subsequent therapy. The patient achieved partial response only after three daratumumab doses.

Pancytopenia is a relatively uncommon finding of MM [5]. Its pathogenesis is largely explained by replacement of the bone marrow with myeloma cells. Treatment strategy in this setting is complicated because even novel agents can further aggravate cytopenias [6]. In the current case, daratumumab efficiently eradicated myeloma cells, thereby inducing reconstitution of normal hematopoiesis. Daratumumab may serve as a promising treatment option for MM with cytopenias.

On the other hand, prolonged severe neutropenia was caused by a single daratumumab dose. Pretreatment bone marrow hypoplasia may be associated with this adverse event. Close monitoring of hematotoxicity and optimization of treatment schedule are required when MM patients with low baseline ANC undergo daratumumab therapy. G-CSF primary prophylaxis may be useful to reduce the risk of infections [6].

We should be reminded that neutropenia does not always predict favorable treatment outcomes. According to a phase 2 trial of daratumumab monotherapy, the incidence of grade 3–4 neutropenia was similar between responders and non-responders [2]. A large-scale study is warranted to further clarify the efficacy and safety profiles of daratumumab for patients with low baseline ANC.
